# Polypiperazine-Based Micelles of Mixed Composition for Gene Delivery

**DOI:** 10.3390/polym16213100

**Published:** 2024-11-04

**Authors:** Rumena Stancheva, Emi Haladjova, Maria Petrova, Iva Ugrinova, Ivaylo Dimitrov, Stanislav Rangelov

**Affiliations:** 1 Institute of Polymers, Bulgarian Academy of Sciences, “Akad. G. Bonchev” St., Bl. 103-A, 1113 Sofia, Bulgaria; rstancheva@polymer.bas.bg (R.S.); dimitrov@polymer.bas.bg (I.D.); 2 Institute of Molecular Biology, Bulgarian Academy of Sciences, “Akad. G. Bonchev” St., Bl. 21, 1113 Sofia, Bulgaria; mhristova84@abv.bg (M.P.); ugryiva@gmail.com (I.U.)

**Keywords:** gene/DNA delivery, polyplexes, micelleplexes, cationic copolymers, PEO-PPO-PEO copolymers, mixed polymeric micelles, internalization/transfection

## Abstract

We introduce a novel concept in nucleic acid delivery based on the use of mixed polymeric micelles (MPMs) as platforms for the preparation of micelleplexes with DNA. MPMs were prepared by the co-assembly of a cationic copolymer, poly(1-(4-methylpiperazin-1-yl)-propenone)-b-poly(d,l-lactide), and nonionic poly(ethylene oxide)-b-poly(propylene oxide)-b-poly(ethylene oxide) block copolymers. We hypothesize that by introducing nonionic entities incorporated into the mixed co-assembled structures, the mode and strength of DNA binding and DNA accessibility and release could be modulated. The systems were characterized in terms of size, surface potential, buffering capacity, and binding ability to investigate the influence of composition, in particular, the poly(ethylene oxide) chain length on the properties and structure of the MPMs. Endo–lysosomal conditions were simulated to follow the changes in fundamental parameters and behavior of the micelleplexes. The results were interpreted as reflecting the specific structure and composition of the corona and localization of DNA in the corona, predetermined by the poly(ethylene oxide) chain length. A favorable effect of the introduction of the nonionic block copolymer component in the MPMs and micelleplexes thereof was the enhancement of biocompatibility. The slight reduction of the transfection efficiency of the MPM-based micelleplexes compared to that of the single-component polymer micelles was attributed to the premature release of DNA from the MPM-based micelleplexes in the endo–lysosomal compartments.

## 1. Introduction

Nucleic acid delivery has been an active research field over the past two decades. The developments in the field have recently been accelerated by the successful application of RNA-based systems, particularly, vaccines to combat the SARS-CoV-2 pandemic [1]. The recent advancements in nucleic acid (RNA, DNA) delivery provoked great preclinical and clinical progress, opening new horizons for developing therapies for a range of diseases including infectious diseases, cancer, and genetic diseases. Due to their susceptibility to degradation in the extracellular environment and difficulties in crossing cell membranes, nucleic acids typically require a delivery vehicle to protect them from enzymatic degradation, promote cellular uptake, and overcome a number of extra- and intracellular obstacles. Despite the success of approved therapies [2,3] based on viral vectors [4,5,6], this approach is often associated with a number of potential risks for patients (high cytotoxicity, severe immune response, insertional mutagenesis, and restoration of virulence resulting in additional diseases) [2,3,7]. Non-viral vectors [8,9] are generally regarded as safer delivery systems compared to viral vectors. In addition, they display significant advantages and benefits, related to the ease of chemical modification, larger payload capacity, and possibilities for low-cost large-scale production. A number of non-viral systems of different nature have been developed so far, including lipids [10,11], liposomes [12,13], polymers [14,15,16,17] and polymer-based particles [18,19], and inorganic materials [20,21]. Some of the most extensively studied nucleic acid delivery systems are based on cationic polymers such as chitosan [22], polyethyleneimine [23,24], poly(L-lysine) [25,26], and poly(2-dimethylaminoethylmethacrylate) [27,28,29]. They can compact nucleic acids through electrostatic interactions to form polyplexes and shield their negative charge to enhance cellular uptake [30]. Ideally, the copolymers should react upon pH changes in the endosome with protonation, which eventually induces endosomal escape via the so-called *proton sponge* effect and release of the polyplex cargo in the cytosol.

Cationic polymers can be integrated into more complex polymer architectures as polycationic moieties in linear block copolymers or side chains of graft copolymers. The combination with appropriately hydrophobic chains/moieties results in the formation of amphiphilic copolymers, which self-assemble in aqueous solution into cationic micelles that can further interact with nucleic acids to form the so-called *micelleplexes*. The latter has been shown to exhibit a number of attractive features and potential to serve as nucleic acid delivery vehicles, as follows [31,32]: good stability in biological fluids [31,32,33], tunable dimensions in the nanoscale [33], core–corona structure allowing for additional loading with hydrophobic drugs or biologically active substances in the core, and increased positive charge density in the corona providing enlarged nucleic acid payload capacity. In addition, the proper copolymer design introducing biodegradable moieties in the micellar core could be beneficial for nucleic acid release and effective clearance from the human body.

**Figure 1:**
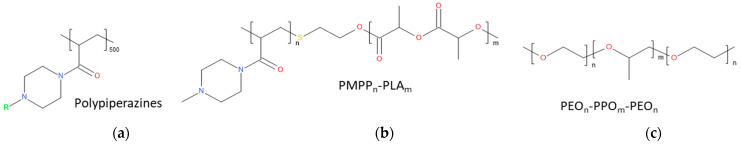
Chemical structures of (**a**) polypiperazine homopolymers according to ref. [34] (R = CH_3_, C_2_H_5_, C_3_H_7_, C_4_H_9_); (**b**) PMPP-PLA and (**c**) PEO-PPO-PEO block copolymers. n and m values represent the degree of polymerization of hydrophilic and hydrophobic blocks of the copolymers and are given in Table S1.

Recently Hausig-Punke et al. [34] investigated a series of charge neutral at physiological conditions polypiperazine homopolymers (Figure 1a) for designing soluble polymeric vectors. Although most of these polymers were only barely charged at physiological pH and strong interaction with genetic material was not expected, the authors demonstrated effective binding of plasmid DNA and high transfection efficiency. Surprisingly, plasmid DNA was found to bind differently than other known transfection polymers (e.g., linear polyethyleneimine), as revealed by a quantitative polymerase chain reaction (qPCR) allowing for the amplification of plasmid DNA within the polypiperazine-based polyplexes. The different binding modes involving interactions with the solvophobic portions of the pDNA but leaving the latter freely accessible made the authors assume an alternative transfection mechanism where a pDNA release is not necessary [34]. A preceding paper also testified for the efficient binding of block copolymers of polypiperazine and polylactide (Figure 1b) with DNA and the formation of micelleplexes that showed promise for gene delivery and regulation [35]. A picture that was derived from the structure of the micelleplexes was for DNA molecules deeply “buried” in the corona of the micelles adopting a flat conformation close to the core–corona interface [35]. Such a construct with seemingly strong interactions might have difficulties with unpacking and release of DNA, which is described as a critical factor in transfection. Furthermore, assuming endocytic uptake, one may expect increased DNA condensation at decreasing pH in the endo/lysosomes, which would further strengthen the binding and, accordingly, disfavor the release of DNA.

Intrigued by the potential of piperazine-based (co)polymers, in this work, we introduce a novel concept of using mixed polymeric micelles (MPMs) as non-viral vector systems. The appropriate design of non-viral vector systems based on mixed polymeric micelles (MPMs) has been found as a successful approach for improving and optimizing the useful properties of single-component polymeric micelles (SCPMs) without the need for complicated synthetic procedures [36,37]. The mixed particles were formed by the co-assembly of the polypiperazine-based cationic block copolymer, poly(1-(4-methylpiperazin-1-yl)-propenone)-b-poly(d,l-lactide) (PMPP-PLA) (Figure 1b), with nonionic poly(ethylene oxide)–b-poly(propylene oxide)–b-poly(ethylene oxide) (PEO-PPO-PEO) block copolymers (Figure 1c). By introducing additional entities incorporated in the mixed co-assembled structures, we aimed to modulate the mode and strength of DNA binding and regulate the accessibility and release of DNA. Enhanced long-term colloidal stability, increased protection from enzymatic degradation, reduced undesired serum interactions and cytotoxicity as well as lowered costs of production are the new functionality/properties/advantages that are produced.

## 2. Materials and Methods

### 2.1. Materials

Tetrahydrofuran (THF, ≥99.9%), ethidium bromide (EtBr), and DNA sodium salt (salmon sperm, 2000 bp) were received from Sigma-Aldrich (Merck-Bulgaria, Sofia, Bulgaria). The amphiphilic poly(1-(4-methylpiperazin-1-yl)-propenone)-b-poly(d,l-lactide) (PMPP-PLA) block copolymer was synthesized according to a procedure described elsewhere [35]. PEO-PPO-PEO block copolymers *Pluronic* F77®, *Pluronic* P65®, and *Pluronic* L64® were purchased from BASF Corporation, Ludwigshafen am Rhein, Germany. The chemical structures and molecular characteristics, composition, and degrees of polymerization of the block copolymers are presented in Figure 1 and Table S1, respectively. For the preparation of DNA solutions and polymer dispersions, ultra-pure water (>18 MΩ) was used.

#### 2.1.1. Cell Lines

H1299, a cell line derived from human non-small-cell lung cancer, and L929, a mouse fibroblast cell line, were purchased from ATCC (LGC Standards, Kiełpin, Polond) and cultured under conditions of 37 °C, 95% air, and 5% CO_2_. RPMI-1640 (Thermo Fisher Scientific, Antisel, Sofia, Bulgaria) and Eagle’s Minimum Essential Medium (Thermo Fisher Scientific, Antisel, Sofia, Bulgaria) served as the base mediums for H1299 and L929, respectively. Complete growth media for both cell lines were prepared by supplementing with 10% fetal bovine serum (Thermo Fisher Scientific, Antisel, Sofia, Bulgaria) and a solution containing penicillin, streptomycin, and amphotericin B (Merck-Bulgaria, Sofia, Bulgaria).

#### 2.1.2. Preparation of Polymer Micelles

SCPMs were prepared by dropwise addition of a PMPP-PLA block copolymer solution in THF to an appropriate quantity of deionized water to obtain a final concentration of 0.5 mg·mL^−1^. For the preparation of MPMs, appropriate amounts of PMPP-PLA and a *Pluronic*^®^ block copolymer at a molar ratio of 1:1 were dissolved in THF. Then, the mixed copolymer solutions were added dropwise to an appropriate quantity of deionized water to obtain a final concentration of 0.5 mg mL^−1^. Afterward, all dispersions were subjected to extensive dialysis against water using SpectraPore 7 dialysis membranes (MWCO 8000, Repligen, Lund, Sweden) in order to remove the organic solvent. Based on preliminary results, at the molar ratio of 1:1, the effects of introducing *Pluronic*® block copolymers are the most pronounced without strongly influencing or even losing the ability to interact with and bind DNA, packaging capacity, and transfection efficiency of the PMPP-PLA block copolymer.

#### 2.1.3. Preparation of Micelleplexes

The micelleplexes were formed in water at neutral pH and ambient temperature. Appropriate amounts of micellar dispersions (0.5 mg mL^−1^) and DNA (0.1 mg mL^−1^) solutions were mixed to give amino-to-phosphate group ratios (N/P) in the 4–25 range. Micelleplexes with GFP-encoding plasmid DNA (pEGFP-N1) were prepared at a N/P ratio of 10 following the same procedure.

### 2.2. Methods

#### 2.2.1. Dynamic and Electrophoretic Light Scattering (DLS and ELS)

DLS and ELS were performed on a NanoBrook 90Plus PALS instrument (Brookhaven Instruments Corporation, Nashua, NH, USA), equipped with a 35 mW red diode laser (λ = 640 nm). The determination of the hydrodynamic radius, R_h_, was carried out at a scattering angle (θ) of 90° using the equation of Stokes–Einstein (Equation (1)):

R_h_ = kT/6πηD (1)

where k is the Boltzmann constant, η is the solvent viscosity at temperature T in Kelvin, and D is the diffusion coefficient at θ = 90°. Each measurement was performed in triplicate.

The ζ-potential measurements were carried out at a scattering angle (θ) of 15°. The principles of electrophoretic light scattering (ELS) and phase analysis light scattering (PALS) were applied. The ζ potentials were calculated from the obtained electrophoretic mobility using the Smoluchowski equation (Equation (2)).

ζ = 4πηυ/ε(2)

where η is the solvent viscosity, υ is the electrophoretic mobility, and ε is the dielectric constant of the solvent. Each measurement was performed in triplicate.

To follow the behavior of SCPMs, MPMs, and micelleplexes at endo–lysosomal conditions, the pH value of each sample was adjusted to 4 by using 0.1 M HCl. Then, the dispersions were incubated at 37 °C for 24 h and their R_h_ and ζ-potential were determined. Each measurement was performed in triplicate.

#### 2.2.2. Atomic Force Microscopy (AFM)

The AFM images were obtained using a Bruker NanoScope V9 Instrument (Bruker, Billerica, MA, USA) operating at a 1.00 Hz scan rate under ambient conditions. An amount of 2 μL of micellar or micelleplex dispersions was placed onto a freshly cleaned glass substrate (1 cm^2^) and spin-casted at 2000 rpm for a minute. AFM measurements were performed in ScanAsyst mode (Peak Force Tapping, Bruker, Billerica, MA, USA).

#### 2.2.3. Determination of Critical Micellization Concentration (CMC)

CMCs of the PMPP-PLA block copolymer as well as of the blends with the PEO-PPO-PEO block copolymers were determined by dye solubilization method using the hydrophobic dye 1,6-diphenyl-1,3,5-hexatriene (DPH). An amount of 2 mL of micellar dispersions at varying concentrations in the 0.001 to 0.5 mg mL^−1^ range was prepared by diluting a stock solution. Then, 20 μL of the DPH solution in methanol (0.4 mM) was added to each sample. The samples were incubated in the dark at room temperature. After 18 h, UV–VIS absorption spectra of DPH were recorded in the range of λ = 300–500 nm. Spectra were taken on a Beckman Coulter DU^®^ 800 (Beckman Coulter, Brea, CA, USA) spectrophotometer by using a quartz cell with a path length of 1 cm. The CMCs were determined from the break of the absorbance intensity at 356 nm vs. concentration curves.

#### 2.2.4. Buffering Capacity

The buffering capacity of the SCPMs and MPMs was determined by standard acid-base titration. Firstly, the pH of the micellar dispersions (0.5 mg mL^−1^) was adjusted to 10 by using 0.1 M NaOH, and then a fixed volume (2 mL) of each sample was titrated with 0.1 M HCl to reach pH 3 as 10 μL aliquots were added. The pH was measured after each addition. Pure water was titrated as a control.

#### 2.2.5. Ethidium Bromide Quenching Assay and DNA Release

DNA solutions were prepared followed by the addition of EtBr at molar ratio [EtBr] = [P]/4, where [P] is the concentration of DNA phosphate groups. Micelleplexes in the N/P range from 4 to 25 were formed upon the addition of the stained DNA solution to the dispersions of SCPMs and MPMs. The EtBr quenching was followed by fluorescence spectroscopy. Afterward, the micelleplex dispersions were acidified to pH 4 by adding 0.1 M HCl. The fluorescence intensity was measured again after incubation for 24 h at endo–lysosomal conditions (pH 4, 37 °C). The fluorescence of all samples was measured at λ_ex_ = 535 nm and λ_em_ = 600 nm on an Agilent Cary Eclipse (Agilent Technologies, Santa Clara, CA, USA) fluorescence spectrometer using a quartz cell with a path length of 1 cm.

#### 2.2.6. Cytotoxicity Test

The cytotoxicity of the examined micelles and their corresponding micelleplexes was assessed using the MTT test [38]. In this assay, 3 × 10^3^ cells per well from H1299 and L929 cell lines were seeded into 96-well flat-bottomed plates (Corning Costar Flat Bottom Cell Culture Plate, FOT Ltd., Sofia, Bulgaria). After a 24 h incubation period, the cells were exposed to varying concentrations of the investigated samples. The serial dilutions ranged from 4 to 128 μg mL^−1^ for micelles and micelleplexes relative to the concentration of the micelles in the complexes. Following a 72 h incubation at 37 °C and 5% CO_2_, the medium was replaced with phenol red-free medium containing MTT (Invitrogen, Antisel, Sofia, Bulgaria) at a final concentration of 0.5 mg mL^−1^. The cells were then incubated for an additional 2.5 h at 37 °C and 5% CO_2_. The MTT assay relies on the conversion of the tetrazolium dye MTT (3-(4,5-dimethylthiazol-2-yl)-2,5-diphenyltetrazolium bromide) into insoluble purple formazan crystals by NAD(P)H-dependent cellular oxidoreductase enzymes in viable cells. For this purpose, 100 μL DMSO per well was added to dissolve the formed tetrazolium crystals. Measurement at 570 nm was conducted using a Varioskan LUX Multimode Microplate Reader (Thermo Fisher Scientific, Antisel, Sofia, Bulgaria), and the obtained data were processed with GraphPad Prism software v.8 (Dotmatics, San Diego, CA, USA).

#### 2.2.7. Internalization

To examine the cell localization of micelleplexes containing DNA stained with EtBr, 5 × 10^4^ H1299 cells per well were seeded on coverslips in a 24-well plate (Corning Costar Flat Bottom Cell Culture Plate). Following a 24 h period of incubation, the cells were treated with micelleplex dispersions at a final DNA concentration of 1 μg mL^−1^ for 24 h. Subsequently, the cells on the coverslips were fixed with 4% (*v*/*v*) paraformaldehyde in PBS for 10 min at room temperature, followed by three 5 min washes with 1×PBS. Finally, the coverslips were mounted with ProLong Diamond Antifade Mountant (Invitrogen, Thermo Fisher Scientific). Imaging was conducted on a Zeiss AxioImager. Z2 microscope with an EC Plan-Neofluar objective (40×/0.75 Ph 2 M27), equipped with a CCD camera Axiocam 705 mono (Zeiss, Jena, Germany). Images from a minimum of three distinct fields were captured for each experimental condition to facilitate subsequent analysis. All observations were made under consistent conditions, and image processing and calculations were performed using the Fiji image processing package.

#### 2.2.8. Transfection

Then, 1 × 10^4^ H1299 cells were plated in 96-well pale (Corning Costar Flat Bottom Cell Culture Plate, FOT Ltd., Sofia, Bulgaria). After a 24 h incubation, the cells underwent transfection with 0.5 μg pEGFP-N1 using Turbofect (Thermo Fisher Scientific, Antisel, Sofia, Bulgaria) following the manufacturer’s instructions. Alternatively, cells were treated with micelleplexes containing the same plasmid DNA at a final DNA concentration of 0.5–2 μg mL^−1^ for 4, 6, or 12 h. EGFP-N1 expression was analyzed 24 h post-transfection/treatment on a Zeiss Axiovert 200 M using a 10×objective PlanApochromat lens (NA = 0.45, Zeiss, Jena, Germany), equipped with a CCD camera AxioCam MRm. A minimum of three distinct observation fields were captured for subsequent analysis in each experimental condition. All images were captured under identical conditions and processed using the Fiji image processing package.

#### 2.2.9. RT-qPCR

First, the H1299 cells were transfected with pEGFP-N1 using Turbofect (Thermo Fisher Scientific, Antisel, Sofia, Bulgaria) or were treated with micelleplexes containing the same plasmid DNA, as described in Section 2.2.8 on transfection. After 24 h of incubation, total RNA was isolated using a DNA/RNA Extracol kit (EURx Gdańsk, Poland) following the manufacturer’s instructions. The synthesis of cDNA from the obtained total RNAs was carried out with the RevertAid H Minus First-strand cDNA Synthesis Kit (Thermo Fisher Scientific, Antisel, Sofia, Bulgaria) according to the manufacturer’s instructions. Then, the obtained single-stranded cDNAs were used as templates for quantitative real-time PCR (qPCR). A 2x SYBR Green PCR Master Mix (Applied Biosystems by Thermo Fisher Scientific) was used to conduct the qPCR following the manufacturer’s instructions. Determination of the relative mRNA expression level of the EGFP gene was used as confirmation of successful transfection. As an endogenous control, the glyceraldehyde-3-phosphate dehydrogenase (GAPDH) gene was used. All reactions were performed in duplicates from two separate experiments. The relative amount of mRNA of the studied genes was calculated using the Delta–Delta Ct method (ΔΔ Ct method) [39]. The primers sequence we used in qPCR were as follows: for EGFP mRNA: 5′—GAT CAC TCT CGG CAT GGA C—3′ forward and 5′—CAT TTT ATG TTT CAG GTT CAG GG—3′ reverse primers; and for GAPDH mRNA: 5′—CTC TGC TCC TCC TGT TCG AC—3′ forward and 5′—TTA AAA GCA GCC CTG GTG AC—3′ reverse primers. qPCR was performed on a Corbett Rotor-gene 6000 (Qiagen, Hilden, Germany) with the following PCR program: 95 °C for 10 min, then 40 cycles of 95 °C for 15 s, 60 °C for 30 s, and 72 °C for 30 s.

#### 2.2.10. Statistical Analysis

A one-way ANOVA Tukey’s multiple comparisons test was employed to assess the mean ethidium bromide fluorescence intensity in each column against the mean ethidium bromide fluorescence intensity in every other column. Additionally, a one-way ANOVA Dunnett’s multiple comparisons test was utilized to compare the mean of the number of transfected cells versus the total number of cells in each column with the mean of the number of transfected cells versus the total number of cells in the control column (Turbofect). To compare ethidium bromide fluorescence between micelleplexes at different time intervals, a two-way ANOVA Dunnett’s multiple comparisons test was used. Probability values were deemed significant when * *p* < 0.05, ** *p* < 0.01, *** *p* < 0.005, **** *p* < 0.001.

## 3. Results and Discussion

### 3.1. Preparation and Characterization of MPMs

**Figure 2:**
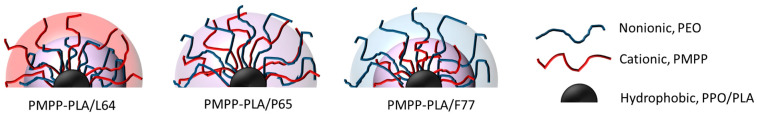
Schematic illustration of the structure of MPMs based on PMPP-PLA and PEO-PPO-PEO block copolymers.

MPMs were formed by co-assembly in water of PMPP-PLA and PEO-PPO-PEO block copolymers at a molar ratio of 1:1 and concentration of 0.5 mg mL^−1^. Three Pluronic copolymers (L64, P65, and F77) were selected for this study. They are characterized by the same or very close degrees of polymerization (DPs) of the middle block of PPO and increase from 13 to 52 DPs of the flanking PEO blocks (see Table S1 for molecular characteristics and other details). Supposedly, the resulting hybrid micelles are of core–corona structure composed of mixed PLA/PPO core and corona built of the hydrophilic PMPP and PEO chains. When the chain lengths of the hydrophilic moieties of the two partners strongly differ, e.g., PMPP-PLA/F77 and PMPP-PLA/L64, the corona might be segregated into two sublayers, as schematically illustrated in Figure 2, giving rise to the formation of onion-like micelles. Due to simple geometrical considerations, the formation of such structures cannot be anticipated if the hydrophilic chain lengths of the two partners are equal/comparable, e.g., the system PMPP-PLA/P65 (Figure 2). SCPMs from the PMPP-PLA block copolymer were prepared for comparative experiments.

The co-assembly process, as well as the formation of mixed particles, is directly related to the hydrophilic–lipophilic balance (HLB) of macromolecules and the critical micellization concentration (CMC) of the partners and their mixtures. The calculated HLB values of the copolymers used reveal the more hydrophobic nature of the PMPP-PLA block copolymer, followed by L64, P65, and F77 (Table S1). The CMCs were determined by a standard dye solubilization method. We used the hydrophobic dye 1,6-diphenyl-1,3,5-hexatriene (DPH), which is effectively solubilized into the hydrophobic domains resulting in a UV absorbance band with a characteristic maximum at 356 nm. The CMCs were determined from the break of the absorbance intensity vs. copolymer concentration curves as shown in Figure S1 and the resulting values are summarized in Table 1. The single break in the dependences (Figure S1), as well as the CMCs that are shifted to higher values with increasing HLB of the PEO-PPO-PEO partner, clearly proved the co-assembly of the two species into MPMs, rather than their separate self-assembly into individual co-existing SCPMs.

The formation of MPMs was followed by dynamic and electrophoretic light scattering. The size and ζ potential distribution plots evidenced the formation of one population of mixed particles. It could be seen from the graphs shown in Figure S2 that monomodal distributions of R_h_ and ζ potential were observed for MPMs. For comparison, physical mixtures (1:1 *v*/*v*) from pre-formed SCPMs of PMPP-PLA and a PEO-PPO-PEO block copolymer invariably exhibited bimodal size and ζ potential distributions (Figure S2) indicating the co-existence of two populations of particles.

Table 1 summarizes the ζ potential and R_h_ values of the MPMs. At physiological pH, the particles are characterized by strongly positive ζ potential and sub-100 nm size. The introduction of L64, that is, the PEO-PPO-PEO copolymer with the shortest PEO chains, hardly influenced ζ potential and R_h_. P65 and F77, however, exerted stronger effects and, as seen from Table 1, ζ potential shifted to less positive values, whereas R_h_ increased by about 6–8 nm. These effects were anticipated and could readily be associated with the longer PEO chains. The polydispersity indexes (PDI) from DLS ranged in the 0.13–0.27 interval, suitable for systemic administration.

AFM was employed to visualize the particles. Representative micrographs of selected dispersions are shown in Figure S3. Seemingly, the monodisperse in size particles of spherical morphology are clearly observable. Their sizes correlate very well with those, determined by DLS. These findings are consistent with the formation of one population of particles of mixed composition.

**Figure 3:**
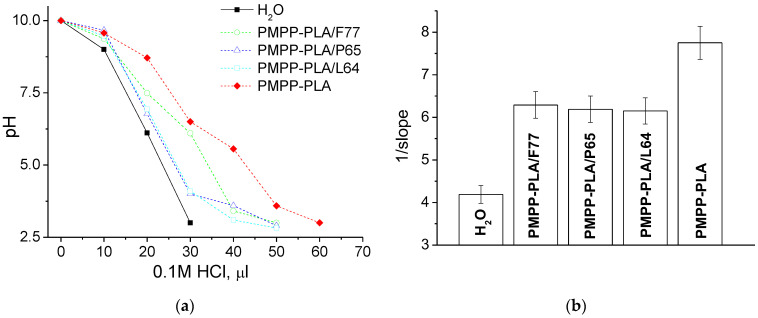
Buffering capacity of SCPMs and MPMs based on PMPP-PLA and PEO-PPO-PEO block copolymers given as (**a**) pH vs. volume of 0.1 M HCl curves and (**b**) reciprocal value of the slope of curves from (**a**).

A desired property and an important characteristic of polymeric vectors is their ability to absorb protons upon pH changes in the endosomal pathway, which then induces endo–lysosomal escape. To estimate the buffering capacity of the investigated micellar systems a standard acid-base titration of micellar dispersions over a pH range from 10 to 3 was performed. Pure water was titrated as a control. The titration curves of PMPP-PLA SCPMs and MPMs are presented in Figure 3a, whereas the intrinsic buffering capability, calculated as 1/slope of the curves, is given in Figure 3b. In contrast to tertiary amines-bearing polymethacrylates such as PDMAEMA with linear [27] or star-like [29] chain architecture, the buffering capacity of the PMPP-PLA SCPMs is comparable to that of the homoPMPP (see also Figure S4a), implying that the effect of the carrier structure (star-like micelles vs. free draining coil) is marginal. Expectedly, the introduction of nonionic PEO-PPO-PEO block copolymers resulted in a moderate (by about 20%) decrease in the protonation ability (buffering capacity) of the MPMs without a pronounced effect of the PEO chain length (Figure 3).

To simulate endo–lysosomal conditions and to follow the changes in the fundamental parameters, the micelles were incubated at pH 4 and 37 °C for 24 h, and their ζ potential and hydrodynamic radii were determined (Table 1). The results showed different behavior of the SCPMs and MPMs with a pronounced effect of the nonionic copolymer in the MPMs. Briefly, the PMPP-PLA SCPMs became strongly protonated (ζ potential reached highly positive values of above 50 mV), but their size practically did not change (Table 1). The latter may indicate that the protonation is not associated with changes in the conformation (elongation, stretching) of the PMPP chains that potentially may contribute to an increase in the overall micellar size. Most probably, although only partially protonated at physiological pH, the PMPP chains have already adopted a maximally elongated/stretched conformation at pH 7. However, the enhanced protonation upon acidification might be related to the enhancement of the hydrophilicity of the PMPP chains. In contrast, for the MPMs, we observed a moderate increase in ζ potential (by about 4.6–10.9 mV), accompanied by a strong increase (23–66%) in the hydrodynamic radius. A possible explanation for this behavior could be conformational changes of the PEO chains from coil or slightly extended conformation to a strongly elongated/stretched state, derived from the loss of mobility at low pH. A similar effect of mixed polymeric corona composed of poly(2-vinylpyridine)/poly(oxyethylene) on R_h_ of micelles prepared from polystyrene-b-poly(2-vinylpyridine)-b-poly(oxyethylene) triblock copolymer was previously observed [40,41]. As the size of the spherical micelles is restricted by the length of the fully stretched copolymer chain, we may also speculate on an increase in the micellar core size that would contribute to the overall size increase. Indeed, the hydrophilization of the PMPP upon acidification as well as radial forces in a direction away from the center that stretching of the PEO chains may exert on the PPO domains could promote loosening of the core and penetration of some water in the PPO domains, ultimately resulting in a micellar core size increase. Notably, the acidification did not bring about to appearance of additional populations of particles and the PDI values remained in the 0.13–0.27 range.

### 3.2. Micelleplexes in Salt-Free Conditions

SCPMs and MPMs were used as platforms for the preparation of micelleplexes in a wide range of N/P ratios varying from 4 to 25. The complexation was performed in aqueous media at pH 7 in order to avoid possible effects provoked by the presence of ions or protons. A commercially available ssDNA of 2000 bp was used for the physicochemical investigations.

**Figure 4:**
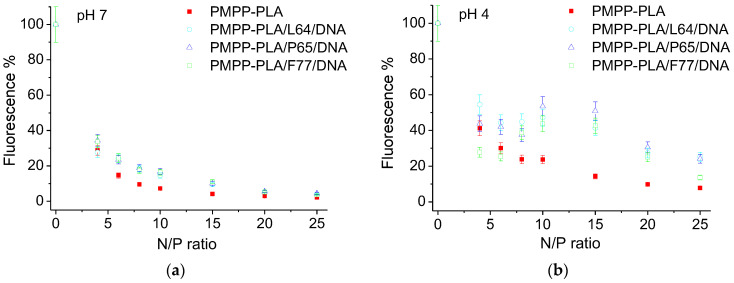
Ethidium bromide fluorescence quenching in micelleplexes formed from SCPMs and MPMs based on PMPP-PLA and PEO-PPO-PEO block copolymers followed at pH 7 (**a**) and after incubation for 24 h in endo–lysosomal conditions (pH 4, 37 °C) (**b**). The fluorescence intensity of ethidium bromide in pure DNA was taken as 100%.

Ethidium bromide (EtBr) quenching assay was used to investigate the binding affinity of the micelles to DNA. When intercalated into DNA bases, EtBr exhibits strong fluorescence, which decreases upon displacement of the dye from the DNA helix. Thus, the complexation ability of the micelles can be evaluated by EtBr fluorescence quenching. Figure 4a shows the profiles of EtBr quenching upon micelleplex formation at different N/P ratios. It is evident that the fluorescence intensity rapidly decreased to about 30% at N/P 4. With increasing N/P the quenching gradually slowed down to reach a reduction of the fluorescence intensity to about 5% and lower indicating almost total displacement of the dye at N/P ratio of 25. Important findings from the fluorescence assay were that (i) the binding affinity of the MPMs, compared to that of the SCPMs, was only slightly reduced by the incorporation of nonionic PEO-PPO-PEO copolymers and (ii) PEO chain length was not a factor that could influence the binding affinity of the MPMs. It is also noteworthy that all differences between SCPMs and MPMs and within the MPMs series of micelleplexes fully disappeared at the highest (20 and 25) N/P ratios.

**Figure 5:**
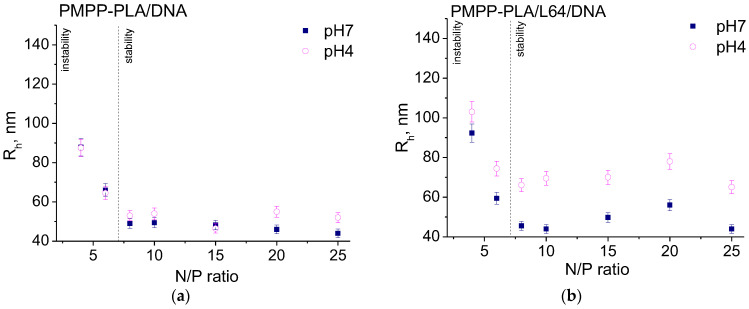

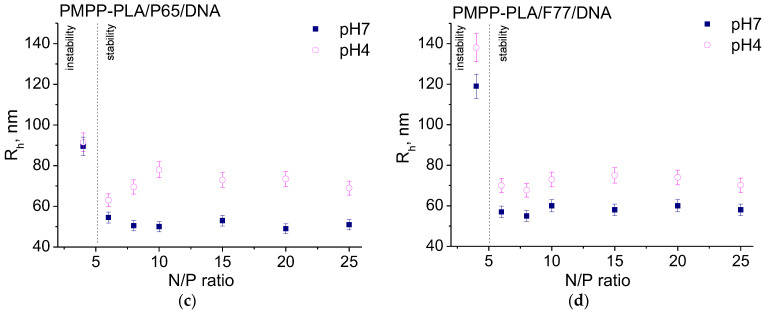
Variations of hydrodynamic radius, R_h_, with the N/P ratio of micelleplexes formed from PMPP-PLA SCPMs (**a**) and PMPP-PLA/L64 (**b**), PMPP-PLA/P65 (**c**), and PMPP-PLA/F77 (**d**) MPMs. Measurements were made at pH 7 (closed symbols) and after incubation for 24 h in endo–lysosomal conditions (pH 4, 37 °C, open symbols).

**Figure 6:**
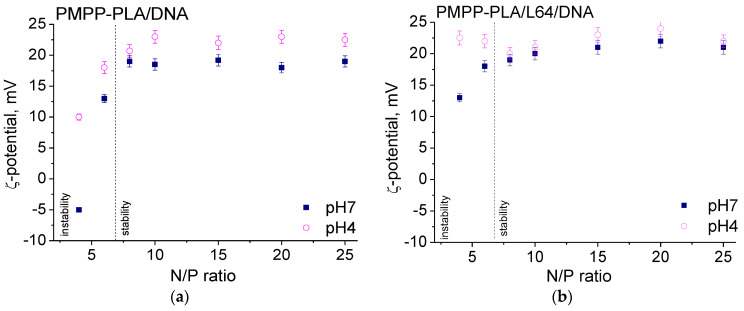

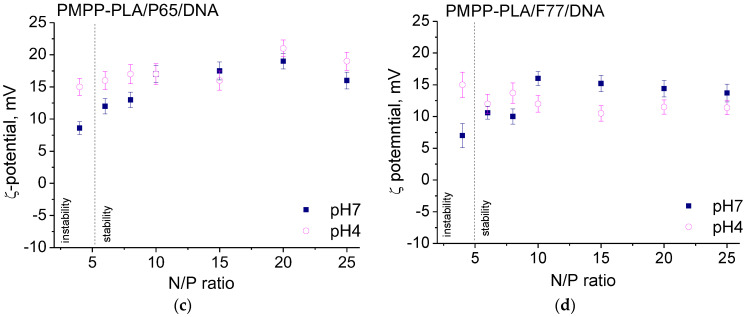
Variations of ζ potential with the N/P ratio of micellplexes formed from PMPP-PLA SCPMs (**a**) and PMPP-PLA/L64 (**b**), PMPP-PLA/P65 (**c**), and PMPP-PLA/F77 (**d**) MPMs. Measurements were made at pH 7 (closed symbols) and after incubation for 24 h in endo–lysosomal conditions (pH 4, 37 °C, open symbols).

The variations of the size and ζ potential of the resulting micelleplexes with the N/P ratio are shown in Figure 5 and Figure 6. At pH 7, the curve patterns of the R_h_ vs. N/P dependences for all investigated systems were identical (closed symbols in Figure 5). Two regions were clearly observable: in the first region, marked as “instability” in Figure 5, the size of the micelleplexes was relatively large (60–120 nm) and rapidly decreased with increasing N/P; the second one (“stability” region in Figure 5) was characterized with constant size values of the micelleplexes that did not depend on the N/P ratio.

Similarly, the ζ potential tended to level off in the “stability” regions to moderately positive values (closed symbols in Figure 6) after rising from negative (SCPMs, Figure 6a) or from less positive (MPMs, Figure 6b–d) in the “instability” regions. The low ζ potential values could be an indicator of the instability of colloidal dispersions. However, precipitation was not observed in the investigated N/P range and dispersions remained colloidally stable for at least a couple of weeks. Thus, the larger micelleplex particles in the “instability” regions (2–3 fold larger than the starting micelles) most probably resulted from the bridging of several micelles by DNA, rather than from incipient instability due to insufficient electrostatic repulsion. Furthermore, the “instability” regions were somewhat wider for the SCPMs and MPMs of PMPP-PLA/L64 (the PEO-PPO-PEO copolymer with the shortest PEO chain), which implied that the longer PEO chains impart a stabilizing effect in terms of lower content needed to achieve colloidal stability. In a recent work, Jiang et al. [42] have observed a similar effect provoked by the addition of a hydrophilic nonionic block as micellar corona. Thus, the increase in nonionic block length effectively decreased the bridging between the micelles and enhanced the colloidal stability of the resulting micelleplexes.

**Figure 7:**
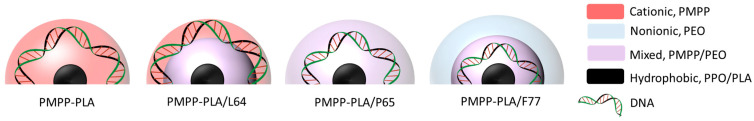
Schematic illustration of the structure of micelleplexes prepared from SCPMs and MPMs based on PMPP-PLA and PEO-PPO-PEO block copolymers.

In the “stability” regions, certain differences between the size of the micelleplexes and that of the corresponding pristine micelles were observed. By certifying the R_h_ values from Table 1 and those from Figure 5 (closed symbols), it is immediately seen that the micelleplexes are slightly but sustainably larger than the corresponding micelles. As the differences were minor but beyond the SD and not negligible, the general impression is that the size of the micelleplexes is governed by the size of the pristine micelles. The ζ potential, however, was more strongly influenced and its reduction to less positive values compared to the corresponding pristine micelles was pronounced (cf. Table 1 and Figure 6, closed symbols). Undoubtedly, these are effects of the complexation of DNA with the micelles and its predominant localization in the corona—in the outer sublayer (PMPP-PLA/L64), in the inner sublayer (PMPP-PLA/F77), in the mixed cationic–nonionic corona (PMPP-PLA/P65) as depicted in Figure 7, or the entirely cationic corona of the PMPP-PLA SCPMs. Apparently, the differences in the composition of the corona and its structure resulting from the different chain lengths of the PEO chains bring about effects with varying impacts on the size and ζ potential.

AFM was used for the visualization of micelleplexes observed in the stability region. The pictures shown in Figure S5 evidenced the presence of well-distributed spherical in shape particles with a size of about 55 nm, which was in very good agreement with the DLS data.

### 3.3. Micelleplexes in Endo–Lysosomal Conditions

Next, we investigated the behavior of the micelleplexes in endo–lysosomal conditions. The fluorescence intensity of the initial dispersions was measured after incubation at pH 4 and 37 °C for a period of 24 h, hypothesizing that the displacement of EtBr is reversible and, when DNA was released, the dye would intercalate between the bases that would produce an increase in the fluorescence intensity. Thus, by following the changes (increase) in the fluorescence intensity, we judge the (extent of) DNA release from the micelleplexes.

Upon incubation, the fluorescence intensity invariably increased for all investigated systems, which resulted in distinctly different profiles of the fluorescence vs. N/P ratio dependences (cf. Figure 4a,b). The increase in fluorescence did not exceed 18% for the micelleplex based on the SCPMs, whereas it was significant, reaching about 40% at certain N/P ratios, for the micelleplexes of the MPMs, indicating the role of the PEO-PPO-PEO copolymer in the DNA release. Variations, although slight but noticeable, were observed among the micelleplex series based on the MPMs, implying the effects of the PEO chain length and, possibly, the structure of the corona and the predominant localization of DNA in the corona. Thus, the systems based on PMPP-PLA/L64 and PMPP-PLA/P65 MPMs exhibited the highest fluorescence intensity increase at the most N/P ratios. An interesting observation was that the increase in the fluorescence intensity went through maxima at N/P ratios of 10 and 15, implying the importance of the DNA content in the micelleplexes.

The size of the micelleplexes based on the SCPMs of PMPP-PLA was practically not influenced by the changes in the environmental conditions (Figure 5a) and their ζ potential was slightly shifted to more positive values (Figure 6a). Just the opposite was observed for the micelleplexes based on MPMs: R_h_ significantly increased, giving rise to the formation of hysteresis in the R_h_ vs. N/P dependences at pH 7 and pH 4 (Figure 5b–d), whereas the variations in ζ potential were very small and frequently negligible (Figure 6b,c). Surprisingly, the hystereses were larger (larger increase in R_h_) for the PMPP-PLA/L64 and PMPP-PLA/P65 micelleplexes, that is, shorter PEO chains (Figure 5b,c), whereas for the PMPP-PLA/F77 micelleplexes (the longest PEO chains) ζ potential, also contrary to the expectations, attained generally less positive values (Figure 6d).

Taken together, the observed findings could be associated with the specific structure and composition of the corona and the localization of DNA. The corona of the SCPMs is entirely built of cationic PMPP chains. In acidic media, more amino groups protonate, thus creating extra sites for electrostatic interactions to which uncomplexed fragments of DNA bind. These events prevented the adoption of a highly stretched conformation of the PMPP chains and a strong increase in ζ potential of the micelleplexes and therefore did not result in any appreciable changes in the size and ζ potential of the SCPMs-based micelleplexes. One can speculate here on the enhancement of the micelleplex strength, resulting from the increasing number of interaction sites and stronger DNA binding.

DNA is predominantly located in the periphery of the mixed (cationic + nonionic) corona of PMPP-PLA/P65 or the mixed outer sublayer of the corona of PMPP-PLA/L64 due to steric hindrance of the nonionic PEO chains (Figure 7). This creates constraints on the PEO chains that are squeezed and confined in a space that is smaller than they would normally occupy. In such conditions, they store energy and exert forces on the DNA-PMPP complex that are counterbalanced by the strength of the complex. Upon incubation at pH 4, the PEO chains lose mobility and undergo a transition to more extended conformation, as described above. They start to push the complex inside out, destroy the balance, and cause detachment of fragments of DNA. Thus, the expanding (swelling) of the inner sublayer (PMPP-PLA/L64) and inner regions (PMPP-PLA/P65) of the corona and partial release of DNA fragments are observable as a substantial increase in R_h_ and very slight, if any, changes in ζ potential.

DNA is located in the inner sublayer of the corona of PMPP-PLA/F77 micelles (Figure 7). The corona is thicker than those of the PMPP-PLA SCPMs and the other two MPMs due to the longer PEO chains of the PEO-PPO-PEO copolymer, which could hinder or retard the release of DNA. The slighter size increase and ζ potential shift to lower values could predominantly but not entirely be related to the conformational changes (transition to more extended conformation) of the PEO chains. Apparently, the release of DNA or DNA fragments takes place as judged from the fluorescence intensity increase (Figure 4b): it involves the mechanism of DNA fragment release described above for the PMPP-PLA/L64 and PMPP-PLA/P65 systems and is somewhat hampered due to the specific structure of the corona.

### 3.4. Biocompatibility of MPMs and Micellplexes

**Figure 8:**
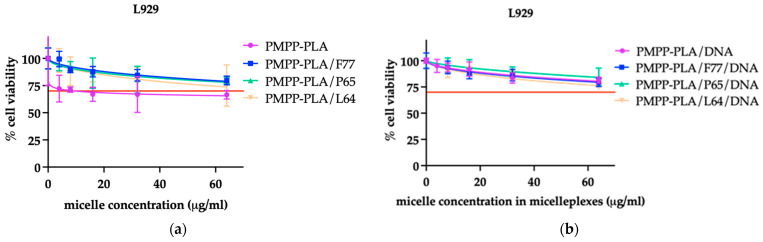
Determination of the cytotoxic effect of the tested micelles (**a**) and their respective micelleplexes (**b**) on L929 cells was conducted by dose–response curves using the “concentration vs. normalized response (variable slope)” algorithm in GraphPad Prism v.8 software. Cell viability is indicated as a percentage relative to untreated control cells. The presented data include the mean ± SD of four replicates from two separate experiments. The red line corresponds to 70% cell viability.

To assess a system as a potential gene delivery platform, essential parameters such as biocompatibility, the capability to release the nucleic acid payload in the appropriate intracellular compartment, and transfection efficiency need to be examined. In order to achieve a therapeutic impact, it is imperative that genetic material maintains stability within the bloodstream, undergoes efficient transport for intercellular functions, and successfully evades the endosome once it enters the cell. Moreover, for plasmid DNA (pDNA) to exert its effects, it must also have the ability to penetrate the nucleus of the cell for gene expression. Therefore, the cytotoxic potential of the delivery systems is of paramount importance [43]. The micelles and micelleplexes were initially examined for their concentration-dependent cytotoxicity, in accordance with the guidelines outlined in ISO 10993-5 [44], utilizing the MTT cell viability assay. Testing was conducted on two cell lines—human malignant cell line H1299 (Figure S6) and non-cancerous murine fibroblast cell line L929 (Figure 8 and Figure S7)—both widely utilized in studies related to gene delivery and transfection. The choice of cell lines H1299 and L929 in this study is grounded in their distinct biological characteristics, which provide a comprehensive evaluation of the polymeric micelles’ biocompatibility and transfection efficiency. Cancer cell lines typically have higher rates of proliferation, which can influence transfection efficiency. Evaluating transfection in H1299 cells helps determine the effectiveness of the micelles in delivering genetic material to rapidly dividing cells, a characteristic of many tumors. Testing on L929 cells helps determine the biocompatibility of the micelles. Since these are non-transformed, non-cancerous cells, they provide a baseline for understanding any cytotoxic effects the micelles might have on normal, healthy cells. Using both human and animal cell lines adheres to standard practices in preclinical research, ensuring that results are robust and applicable across different biological contexts. This dual approach can help in predicting potential in vivo outcomes more accurately.

The experimental results were normalized as percentages relative to the untreated control and subjected to mathematical analysis through nonlinear regression using GraphPad Prism software (Figure 8). A reduction in cell viability of 70% or higher was regarded as indicative of potential cytotoxicity. In that aspect, the present micelles and micelleplexes can be regarded as non-toxic.

Based on this analysis, dose–response relationships were established, and the half-maximal inhibitory concentrations (IC_50_) were determined and documented in Table 2 and (Figures S6 and S7). Notably, no signs of cytotoxicity were observed across all tested micelles and micelleplexes up to a final micelle concentration of 128 μg·mL^−1^. In our experiments, we employed a N/P ratio of 10. At this ratio, to ensure sufficient DNA quantity, micelleplex concentrations of approximately 20 μg mL^−1^ were utilized. Within this concentration range, our systems demonstrated non-toxic characteristics. While higher cytotoxicity was observed in the H1299 cell line (Figure S6 and Table 2), the evaluation of biocompatibility at a micelleplex concentration of 20 μg·mL^−1^ showed no cytotoxicity.

Notably, there was a significant difference in cytotoxicity between the two cell lines, which highlights the varying responses of cancerous and normal cells to the polymeric micelles. The observed differences in cytotoxicity between H1299 and L929 cell lines underscore the importance of evaluating gene delivery systems in both cancerous and normal cell contexts. The higher sensitivity of H1299 cells suggests that the micelles and micelleplexes are more effective against cancer cells, which is desirable for targeted cancer therapies. Meanwhile, the lower cytotoxicity in L929 cells indicates good biocompatibility, making these micelles suitable for potential therapeutic applications.

Although a difference in cytotoxicity was observed between the two tested cell lines, the general trend is clear: all MPMs are less cytotoxic than SCPMs, whether as polymer micelles or micelleplexes. This can be explained by the presence of cationic charges at pH 7.4 and the effect of the nonionic copolymer in the MPMs. As for the difference in cytotoxicity, we presume that it is due to the different origin and cell type (H1299 is a cell line derived from human non-small-cell lung cancer with epithelial morphology, whereas L929 is a normal mouse cell line with fibroblast morphology). It is worth noting that the lowest cytotoxicity is observed in PMPP-PLA/F77 micelles, where the corona is thicker than those of PMPP-PLA SCPMs and the other two MPMs.

All systems are generally non-toxic, as the calculated IC_50_ values consistently surpassed the concentrations utilized for subsequent biological evaluations.

The populations of viable and nonviable cells were also assessed using the trypan blue exclusion assay (see Figures S8 and S9). This assay differentiates between viable and nonviable cells based on membrane integrity. The results were consistent with the MTT assay’s results, showing only a slight, concentration-dependent increase in nonviable cells following incubation with various micelles and micelleplexes.

### 3.5. Micelleplexes Cellular Internalization and Transfection Efficiency

**Figure 9:**
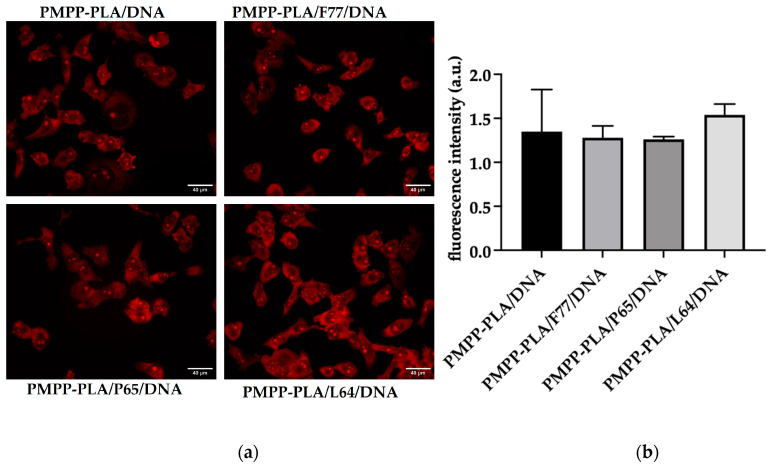
Internalization capacity of tested micelleplexes. (**a**) Representative microscopic images showing H1299 cells treated with the examined micelleplexes at pH = 7, with a final salmon sperm DNA concentration of 1 μg mL^−1^ for 24 h. The scale bar corresponds to 40 μm. (**b**) Quantitative analysis of EtBr fluorescence intensity was conducted using the Fiji image processing package. At least four images for each tested micelleplex from two independent experiments were assessed. The values represent the means ± SD. Statistical analysis was performed using a one-way ANOVA Tukey’s multiple comparisons test to compare the mean of each column with the mean of every other column. No statistically significant differences were observed.

For further evaluation of the SCMP and MPMs, their cellular uptake was studied. Therefore, micelleplexes containing salmon sperm DNA stained with EtBr and then purified from the free dye were applied to H1299 cells seeded on coverslips in OptiMEM medium for 16 h. The uptake of the labeled micelleplexes was investigated via microscopy (Figure 9a). In these experiments, we utilized a DNA concentration in the micellplexes of 1 μg mL^−1^. For quantification of each image, the obtained mean fluorescence intensities were normalized to the number of cells in the captured field (fluorescent objects). As depicted in the bar graph (Figure 9b), there were no statistically significant differences in intensity, indicating good internalization of all investigated systems. It is worth noting, however, that the best internalization was observed for the PMPP-PLA/L64 mixed micelles—the system with the highest ζ potential and smallest hydrodynamic radius (see Figure 5b and Figure 6b). Nevertheless, it should be recalled that this system exhibited the highest cytotoxicity among the studied MPMs but considerably lower than that of the SCPMs (Table 2). The significant decrease in the cytotoxicity barely affecting the cellular internalization is an excellent demonstration of the favorable effects of introducing nonionic PEO-PPO-PEO copolymers in the mixed micelles and length of the PEO chains. It is noteworthy that a diffuse cytoplasmic signal is observed for all systems, suggesting release from endosomes.

Gene delivery systems must effectively transport large nucleic acids such as plasmid DNA across external and internal cellular barriers to reach the nucleus while avoiding side effects [45]. This involves the temporary packaging of pDNA, promoting its cellular uptake, endosomal release, and nuclear entry without harm, even in non-dividing cells. The nuclear envelope poses a significant challenge, especially since efficient gene delivery typically occurs in cells that are actively dividing, where the nuclear envelope breaks down during mitosis. Consequently, non-viral transfections are often cell cycle-dependent, making it difficult to transfect non-dividing cells. This difficulty leads to a continuous introduction of new transfection agents. Despite these efforts, only about 1–10% of the administered pDNA is estimated to reach the nucleus, as shown by various detection methods, indicating that successful nuclear delivery for gene expression remains limited [46]. Fascinated by the impressive uptake and substantial endosomal release, we then examined the transfection efficiency of our systems using commercially available GFP-encoding plasmid DNA (pEGFP-N1). The micelleplexes were prepared at a N/P ratio of 10. The binding efficiency of the SCPMs and MPMs to pEGFP-N1 was evident from the substantial decrease in the fluorescence intensity of EtBr (Figure S10), indicating that SCPMs and MPMs successfully formed complexes. The resulting micelleplex particles were characterized with R_h_ ranging from 60 to 100 nm and moderately positive ζ potential in the 5.1–12.3 mV range (Figure S11).

**Figure 10:**
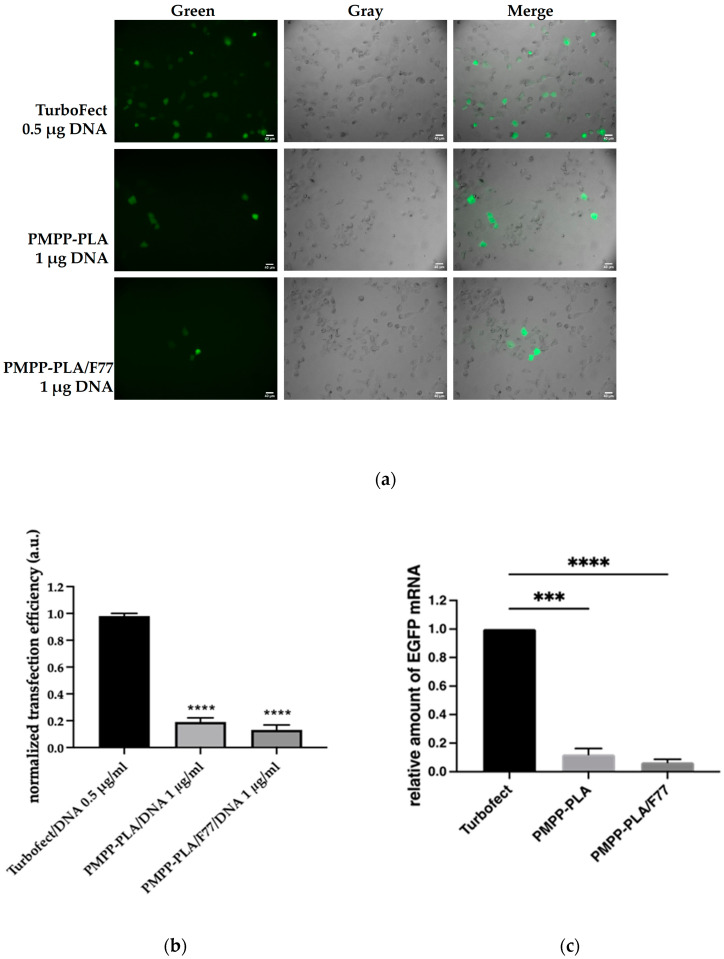
Transfection capacity of tested micelleplexes. (**a**) Representative microscopic images showing H1299 cells transfected with 0.5 μg pEGFP-N1 using Turbofect (positive control) or treated with micelleplexes containing pEGFP-N1 at a final DNA concentration of 1 μg mL^−1^ for 6 h. At least three images for each condition from two independent experiments were assessed. The scale bar corresponds to 40 μm. (**b**) Bar graphs present transfected cells vs. total number of cells from each condition. (**c**) Relative amounts of EGFP gene mRNA after 24 h incubation in H1299 cells transfected with pEGFP-N1 using Turbofect (positive control) or treated with micelleplexes containing the same plasmid DNA. The relative amounts of EGFP gene mRNA in micelleplexes are normalized to the relative amount of EGFP gene mRNA in the positive control (Turbofect). Statistical analysis was performed using a one-way ANOVA Dunnett’s multiple comparisons test to compare the mean of each column with the mean of control column (Turbofect). Probability values indicating significance were considered at the *** *p* < 0.005, **** *p* < 0.001.

Cells exhibiting GFP fluorescence were detected using fluorescence microscopy. In these experiments, various transfection conditions were tested by varying the DNA concentration from 0.5 to 2 μg mL^−1^. Different incubation times with the micelleplexes, ranging from 4 to 12 h, were also examined, along with the following different media: DMEM with 10% FBS, DMEM with 2% FBS, and OptiMEM. The standard transfection agent Turbofect was used as a positive control, and pure plasmid was employed as a negative control. The transfection efficiency was determined by the ratio of transfected cells to the total number of cells in a given field of view. For each condition, at least three separate fields were captured. The best results were observed with transfection in OptiMEM for an incubation period of 6 h with micelleplexes at a DNA concentration of 1 μg mL^−1^ (Figure 10a). No transfection was observed with the pure plasmid and only a few transfected cells were detected with micelleplexes PMPP-PLA/L64 and PMPP-PLA/P65. Successful transfection was achieved with PMPP-PLA SCPMs and PMPP-PLA/F77 MPMs, but both systems demonstrated low relative efficiency, 18% and 10%, respectively, in comparison to Turbofect (Figure 10b).

RT-qPCR quantitatively measures the relative mRNA expression levels of the EGFP gene, providing a direct assessment of the transcriptional activity following transfection. The use of the Delta–Delta Ct method (ΔΔ Ct method) allows for the normalization of EGFP expression to an endogenous control gene (GAPDH), ensuring accurate quantification. On the other hand, immunofluorescence (IF) microscopy detects and visualizes the EGFP protein expression within the cells. The intensity of fluorescence correlates with the amount of EGFP protein present, providing a qualitative and semi-quantitative measure of transgene expression at the protein level Figure 10a,b. The RT-qPCR results show relative amounts of EGFP mRNA in H1299 cells treated with micelleplexes and compare these amounts to the positive control (Turbofect-transfected cells). The level of relative mRNA content indicates successful transfection and robust gene expression (Figure 10c). By estimating the relative levels of EGFP mRNA in micelleplex-treated cells, RT-qPCR validates that the observed fluorescence in IF is due to successful transfection and expression of the transgene. IF experiments include controls to account for autofluorescence, such as unstained cells and cells treated with non-fluorescent micelles. The RT-qPCR data validate the IF data sets by providing quantitative evidence of EGFP mRNA expression, which correlates with the protein expression observed in IF. This multi-level validation ensures the reliability of the transfection efficiency and gene expression results, confirming that the fluorescence signals in IF are due to the successful expression of the transgene delivered by the micelleplexes (compare Figure 10b,c).

The studied MPMs exhibited favorable characteristics, including the optimal size range of 60–120 nm, robust stability of the polymer–DNA complexes indicating protection of DNA from degradation, efficient cellular uptake, and successful endosomal escape. Despite these positive attributes, the transfection efficiency remains modest. A plausible explanation could be their specific structure taken together with the premature release of DNA in the endosomes (see Figure 7 and the discussion in Section 3.3), which could effectively decrease the quantity of the administrated pDNA that reaches the nucleus. Among the MPMs-based micelleplexes, PMPP-PLA/F77 exhibited somewhat retarded DNA release in the endolysosomal conditions (Figure 4b), which appeared to be beneficial, as the PMPP-PLA/F77 micelleplexes exhibited the best transfection efficiency results. In brief, it is conceivable that the incorporation of a PEO-PPO-PEO copolymer further enhances system stability, potentially impeding the accessibility of pDNA to the transcriptional machinery especially at systems based on PMPP-PLA/L64 and PMPP-PLA/P65 MPMs.

Building on the already hypothesized idea that the delivery mechanism of the polypiperazines may not entail complete release of the pDNA and possibility of a direct transfer of the polymer-bound pDNA into the nucleus [34], we may speculate that the enhancement of the strength of the micelleplex based on the PMPP-PLA SCPMs (Section 3.3) might impede the undesired, preliminary release of DNA in the endosomes in contrast to the MPMs-based systems.

## 4. Conclusions

This contribution first examines the structure of MPMs composed of a cationic block copolymer and nonionic PEO-PPO-PEO copolymers. The cooperative process of co-assembly of different in-nature copolymer chains into sub-100 nm core–corona micelles with a specific structure and composition of the corona, governed by the PEO chain length, was unambiguously proved. The variations of the particle characteristics compared to those of the reference SCPMs—a shift of the ζ potential to less positive values, moderate size increase, and reduction of buffering capacity—were associated with the length of the PEO chains. The effects of the introduction of PEO-PPO-PEO copolymers were more visible upon acidification, thereby simulating endo–lysosomal conditions. They were associated with conformational changes (elongation) of the PEO chains and overall hydrophilization of the structures leading to a substantial particle size increase and a moderate rise in the ζ potential. Notably, the binding affinity of the MPMs to DNA was hardly influenced by the incorporation of PEO-PPO-PEO copolymers: compared to the SCPMs, a slight reduction without a pronounced effect of the PEO chain length was documented for all investigated systems. The PEO chain length, however, had a determining role in the localization of DNA in the corona and strongly influenced the fundamental characterizing parameters and behavior of the micelleplexes. Complexation studies based on the EtBr assay indicated that the release of DNA from the micelleplexes based on the MPMs in simulated endo–lysosomal conditions outperformed that of the SCPMs. Although the release of DNA is considered vital for efficient transfection, the MPM-based micelleplexes were less efficient than those based on the SCPMs. Apparently, the superposition of reduced buffering capacity and enhanced DNA release in endo–lysosomal conditions was not favorable and resulted in a lowering of transfection efficiency of the MPM-based micelleplexes. Nevertheless, the MPMs of PMPP-PLA/F77 enable efficient transfection and can be considered promising materials for nucleic acid delivery. They offer low positive net charge at physiological conditions (which may help overcome undesired serum interactions and reduce cytotoxic effects), steric stabilization and stealth properties introduced via PEO chains (improving colloidal stability and enhancing longevity), and a core compartment (allowing for the additional loading of drugs and the formation of co-delivery vehicles). The concept of using MPMs as non-viral vector systems is obviously feasible, which is the main conclusion of this study. Considering the versatile and straightforward preparation of MPMs, countless combinations of copolymers can be formulated in which cationic, nonionic, and hydrophobic blocks can be varied. Furthermore, the integration of additional (third) components bearing targeting moieties also seems conceivable. Possible limitations of the approach could be the strong thermodynamic incompatibility between the blocks, which would prevent the formation of hybrid, mixed structures as well as the unproper design and selection of the constituent copolymers.

## Figures and Tables

**Table 1:** Critical micellization concentration (CMC), ζ potential, and hydrodynamic radius (R_h_) values of SCPMs and MPMs based on PMPP-PLA and PEO-PPO-PEO block copolymers. Measurements were performed at 25 °C.

Micellar Composition	CMC, Mg·mL^−1^	ζ Potential, mV		R_h_, nm	
		pH 7	pH 4	pH 7	pH 4
PMPP-PLA	0.0117 ^a^	31.8 ± 5.2	52.3 ± 2.4	39.8 ± 1.1	40.3 ± 3.2
PMPP-PLA/L64	0.0183	32.2 ± 6.3	38.4 ± 1.9	37.7 ± 2.3	46.5 ± 4.1
PMPP-PLA/P65	0.0214	27.7 ± 5.6	38.6 ± 1.2	47.6 ± 2.8	67.8 ± 5.8
PMPP-PLA/F77	0.0305	23.1 ± 6.1	27.7 ± 2.3	46.2 ± 2.6	76.8 ± 6.1

^a^ Data taken from ref. [35].

**Table 2:** Cytotoxicity of SCPMs and MPMs was determined by the MTT-dye reduction assay after 72 h of continuous exposure to L929 and H1299 cell lines. GraphPad Prism 8 software was used for the calculation of IC_50_ values.

Micelles	IC_50_ Value [μg mL^−1^]	Micelleplex	IC_50_ Value [μg mL^−1^]
**L929**			
PMPP-PLA	161.2 ± 6.2	PMPP-PLA/DNA	398 ± 5.7
PMPP-PLA/F77	642.6 ± 8.4	PMPP-PLA/F77/DNA	894.8 ± 6.9
PMPP-PLA/P65	798 ± 5.9	PMPP-PLA/P65/DNA	808.4 ± 8.6
PMPP-PLA/L64	357.6 ± 7.3	PMPP-PLA/L64/DNA	475.8 ± 9.5
**H1299**			
PMPP-PLA	49 ± 3.4	PMPP-PLA/DNA	23.1 ± 3.8
PMPP-PLA/F77	86.2 ± 5.7	PMPP-PLA/F77/DNA	88 ± 4.2
PMPP-PLA/P65	77.7 ± 2.6	PMPP-PLA/P65/DNA	44.1 ± 2.6
PMPP-PLA/L64	57.6 ± 4.1	PMPP-PLA/L64/DNA	37.1 ± 5.4

## Data Availability

Data are contained within the article.

## Supplementary Materials

The following supporting information can be downloaded at: https://www.mdpi.com/article/10.3390/polym16213100/s1, Table S1: hydrophilic–lipophilic balance, degree of polymerization of hydrophilic and hydrophobic blocks, and critical micellization concentration of PMPP-PLA and PEO-PPO-PEO (Pluronic) block copolymers; Figure S1: CMC plots of MPMs based on PMPP-PLA and Pluronic block copolymers; Figure S2: size and ζ-potential distribution plots of SCPMs, their physical mixture and MPMs prepared at molar ratio 1:1; Figure S3: representative AFM images of PMPP-b-PLA SCPMs and MPMs; Figure S4: buffering capacity of PMPP-PLA micelles, homoPMPP and Pluronic F77, P65, and L64 block copolymers; Figure S5: representative AFM images of micelleplexes prepared from MPMs; Figure S6: the cytotoxic effects against H1299 cells of the tested MPMs and their respective micelleplexes; Figure S7: the cytotoxic effects against L929 cells of the tested MPMs and their respective micellplexes; Figure S8: quantification of viable L929 cells determined after Trypan Blue staining; Figure S9: quantification of viable H1299 cells determined after Trypan Blue staining; Figure S10: ethidium bromide fluorescence quenching in micelleplexes formed from SCPMs and MPMs based on PMPP-PLA and PEO-PPO-PEO block copolymers and pDNA; Figure S11: size and ζ-potential of micelleplexes formed from SCPMs and MPMs and pDNA, at N/P ratio of 10.
