# Supplementary material for: Polypiperazine-Based Micelles of Mixed Composition for Gene Delivery
Source: Polymers (Basel). 2024 Nov 4;16(21):3100. doi: 10.3390/polym16213100 (PMC11548379; doi:10.3390/polym16213100)
Supplement: Supplementary file 1 [file polymers-16-03100-s001.zip › polymers-3250267-SI.pdf]

Supplementary Materials

POLYPIPERAZINE-BASED MICELLES OF MIXED COMPOSITION FOR GENE DELIVERY

Rumena Stancheva[1], Emi Haladjova[1], Maria Petrova[2], Iva Ugrinova[2], Ivaylo Dimitrov[1], Stanislav Rangelov[1]

[1]*Institute of Polymers, Acad. G.Bonchev Str, bl. 103, Bulgarian Academy of Sciences,*

[2]*Institute of Molecular Biology, Acad. G.Bonchev Str, bl. 21, Bulgarian Academy of Sciences*

Table S1. Hydrophilic-lipophilic balance (HLB), degree of polymerization of hydrophilic (n) and hydrophobic (m) blocks and critical micellization concentration (CMC) of PMPP-PLA and PEO-PPO-PEO (Pluronic) block copolymers.

**Copolymer**	**n**	**m**	**HLB**	**CMC, mg/ml**
PMPP-PLA	20	35	7.6	0.0118
Pluronic L64	13	30	7.9	0.0523
Pluronic P65	20	30	10.1	0.0854
Pluronic F77	52	35	13.9	0.1986

For HLB calculation the Griffin method [1] was used following the equation:

$HLB = 20 * M_h/M$

where M_h is the molar mass of the hydrophilic block of copolymer, and M is the molecular mass of the whole macromolecule.

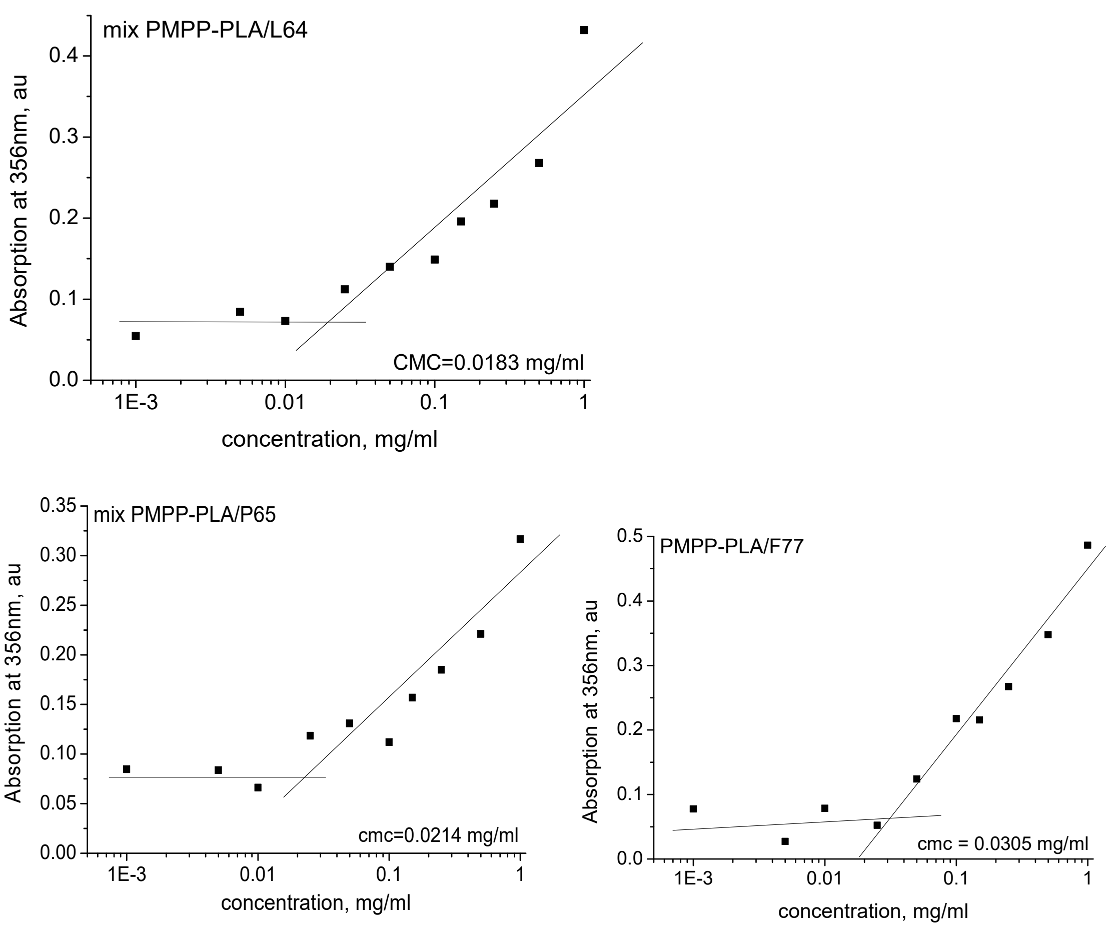

Figure S1. CMC plots of MPMs based on PMPP-PLA and PEO-PPO-PEO block copolymers.

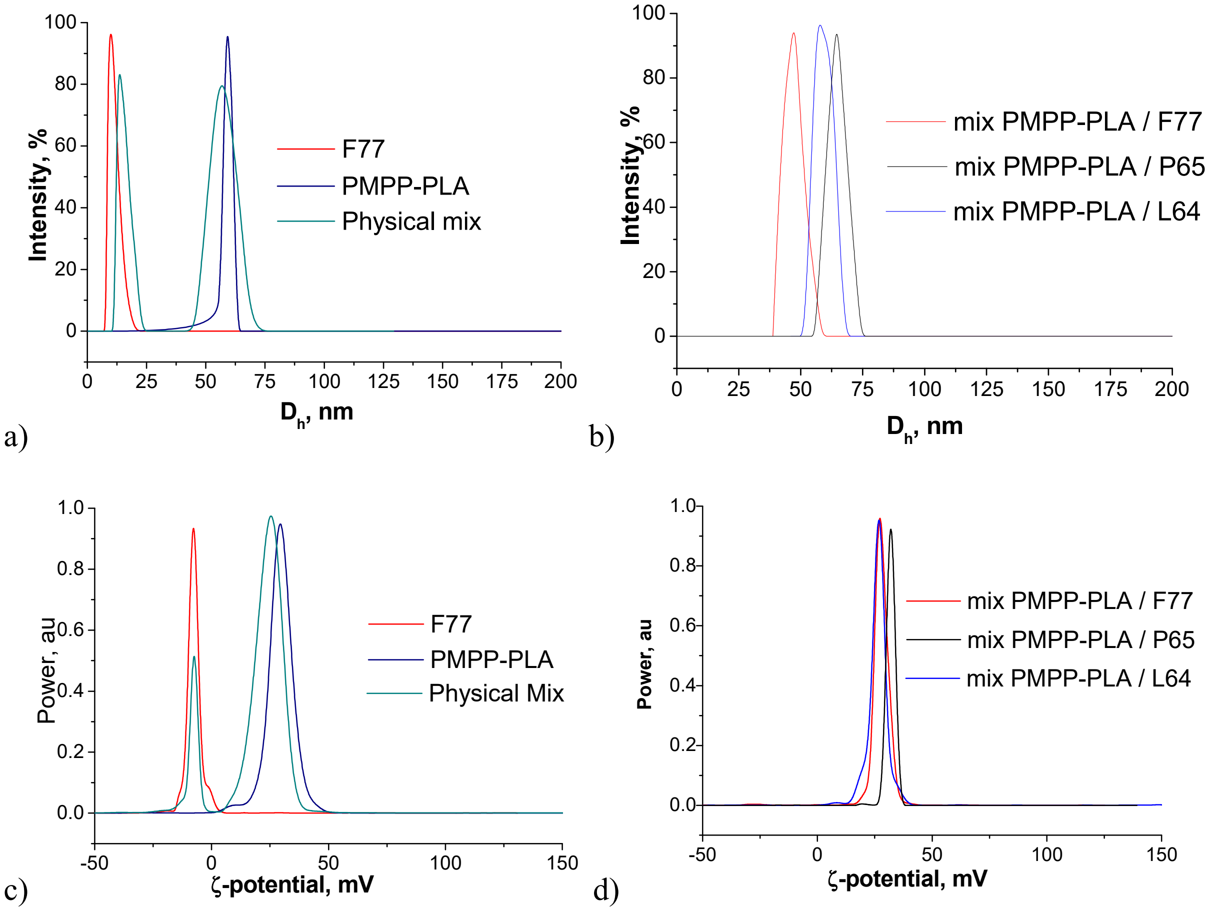

Figure S2. Size (a and b) and ζ-potential (c and d) distribution plots of SCPMs, their physical mixture and MPMs prepared at a molar ratio of 1:1. All measurements were done at concentration of 0.5 $mg.mL^{-1}$ and 25 °C.

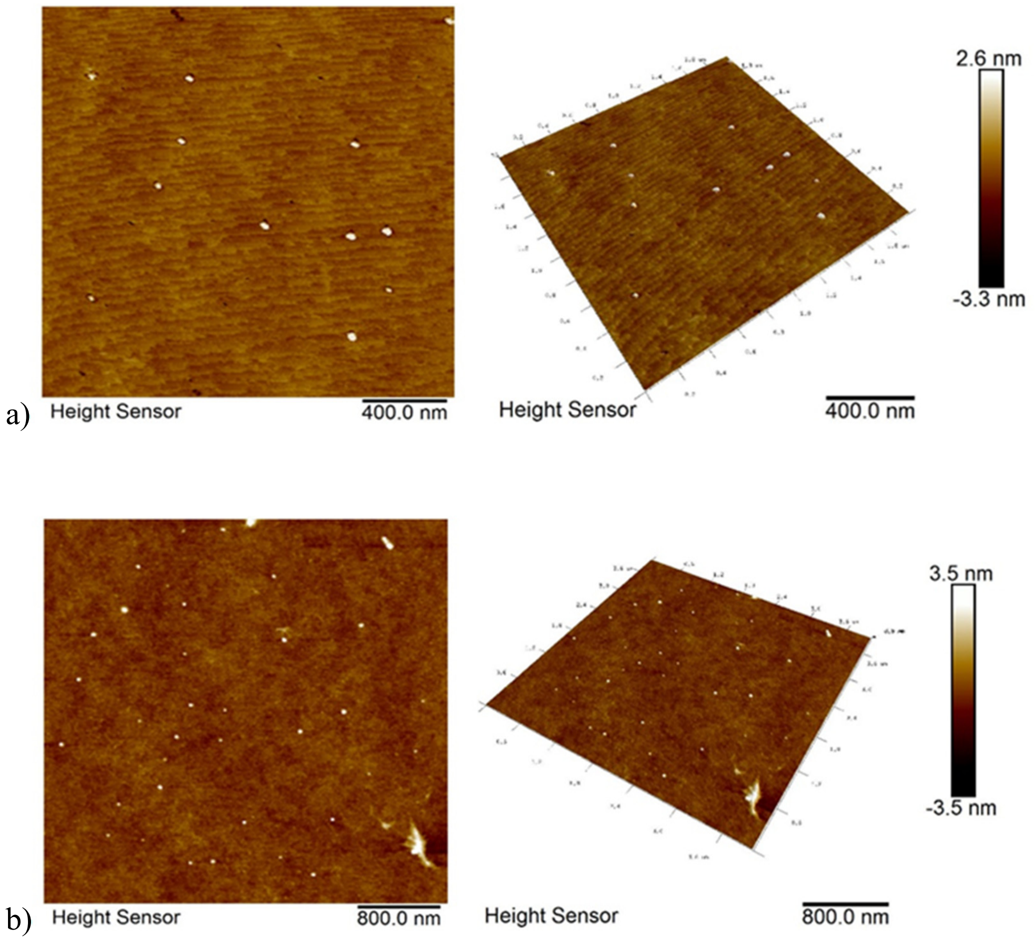

Figure S3. Representative AFM images of (a) PMPP-b-PLA SCPMs and (b) MPMs based on PMPP-b-PLA and Pluronic F77 block copolymers.

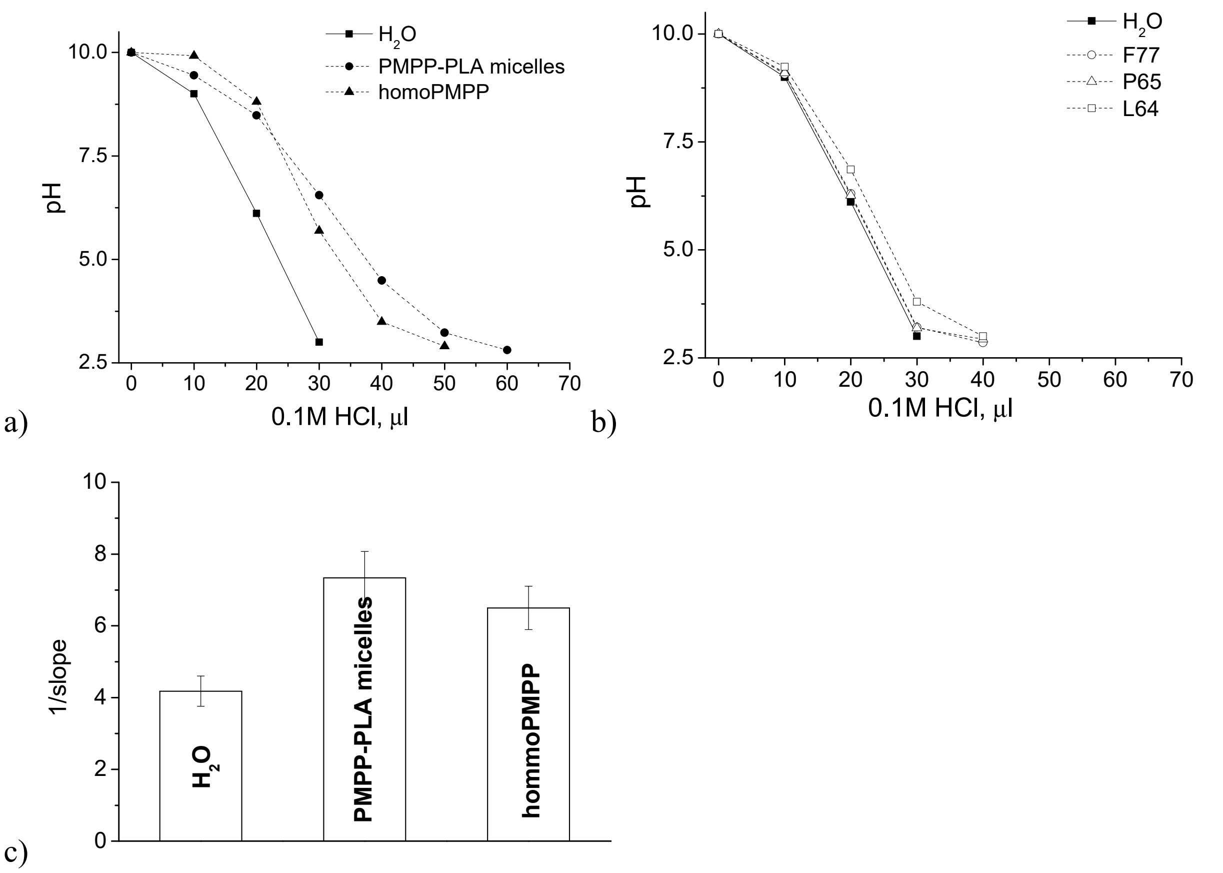

Figure S4. Buffering capacity of a) PMPP-PLA micelles and homoPMPP at concentration of PMPP moieties of 0.105 mg.ml^{-1}; b) Pluronic F77, P65 and L64 block copolymers at concentration of 0.5 mg.ml^{-1} and c) reciprocal value of the slope of curves from (a).

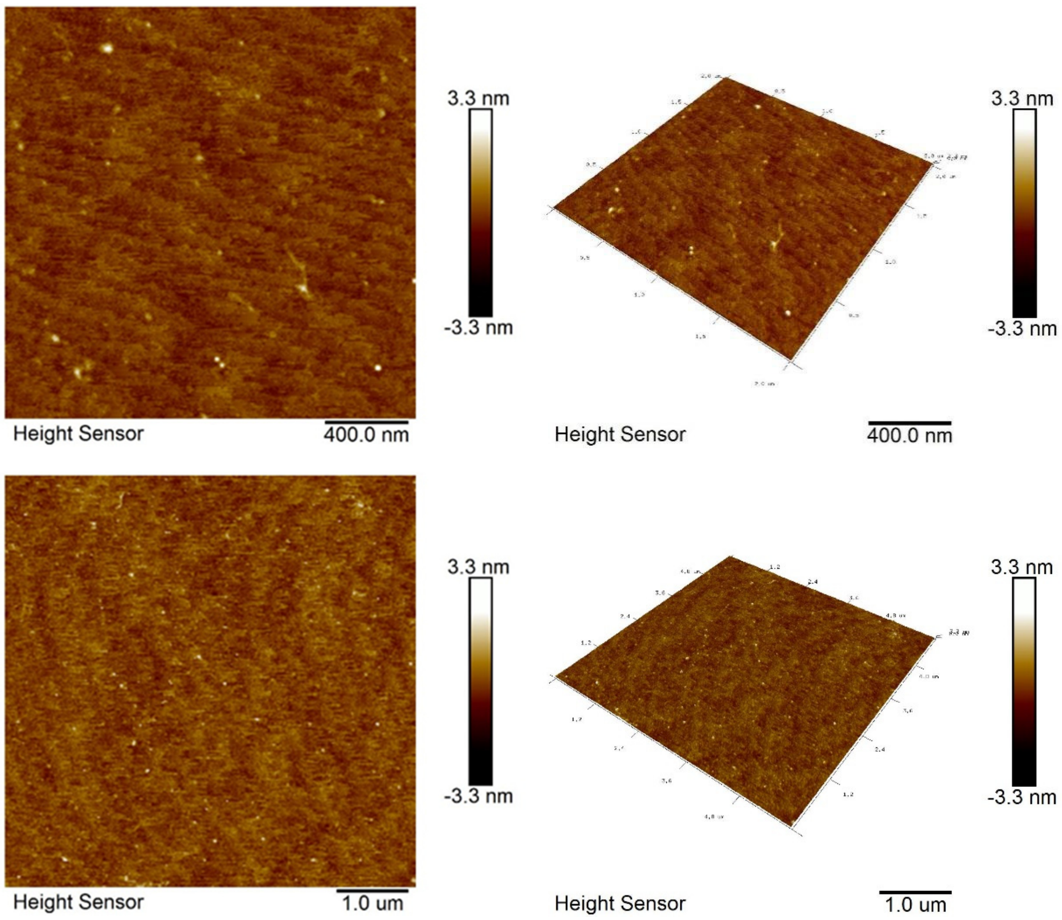

Figure S5. Representative AFM images of micelleplexes prepared at an N/P ratio of 10 from MPMs based on PMPP-PLA and Pluronic F77 block copolymers.

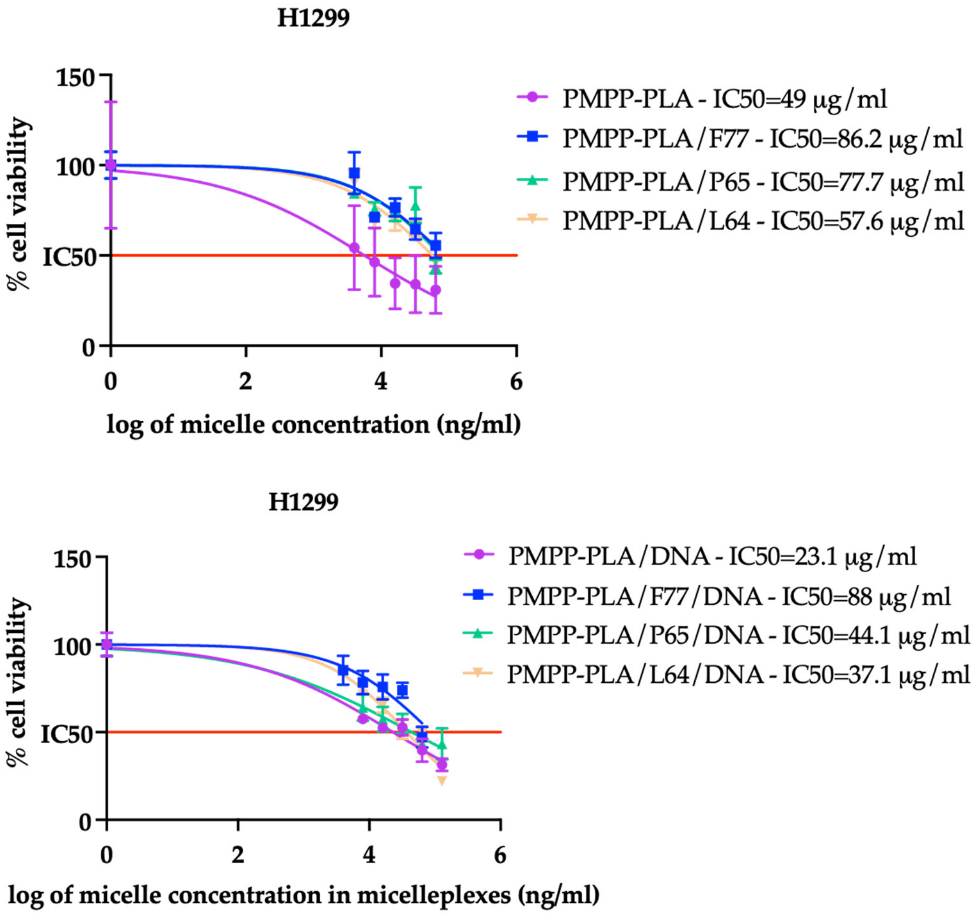

Figure S6. The cytotoxic effects against H1299 cells of the tested SCPMs, MPMs and their respective micelleplexes depicted through dose-response curves utilized for determination of the IC50 values. The curves were generated employing the “log of concentration vs. normalized response (variable slope)” algorithm within GraphPad Prism v.8 software. Cell viability is expressed as a percentage relative to untreated control cells. The presented data include the mean ± SD of four replicates across two separate experiments.

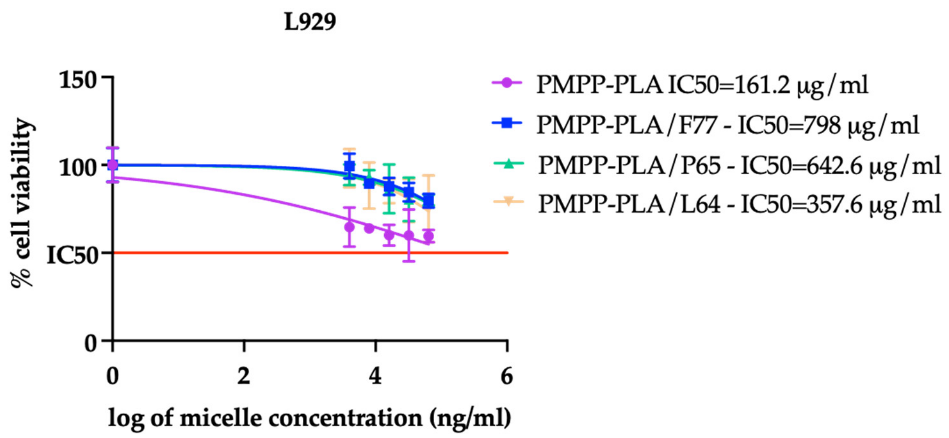

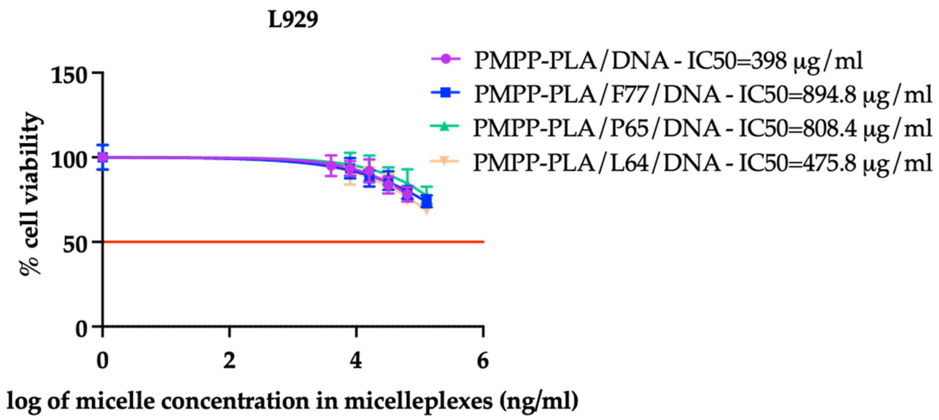

Figure S7. The cytotoxic effects against L929 cells of the tested SCPMs, MPMs and their respective micelleplexes depicted through dose-response curves utilized for determination of the IC50 values. The curves were generated employing the “log of concentration vs. normalized response (variable slope)” algorithm within GraphPad Prism v.8 software. Cell viability is expressed as a percentage relative to untreated control cells. The presented data include the mean ± SD of four replicates across two separate experiments.

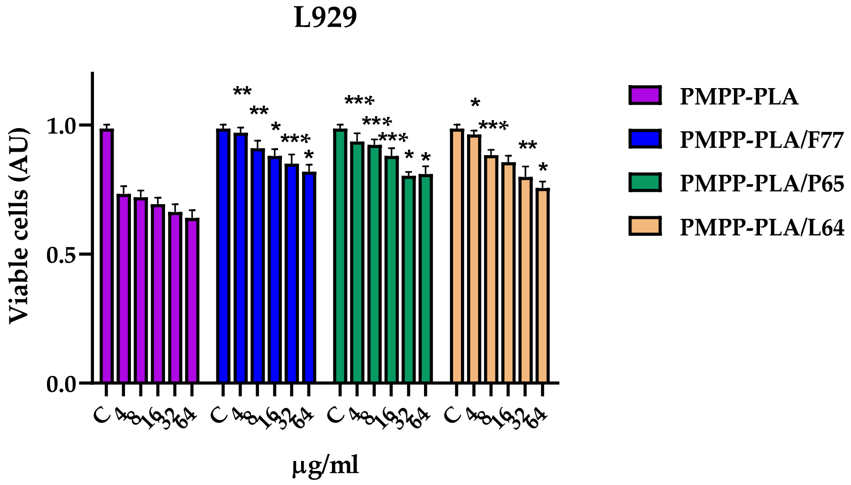

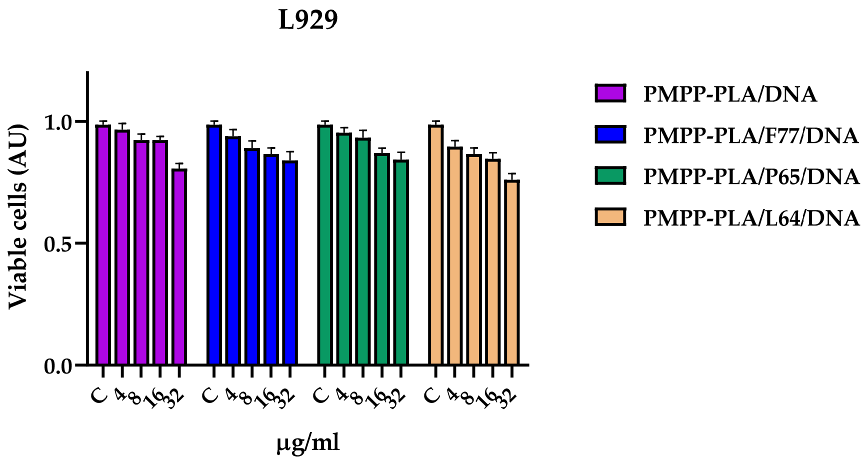

Figure S8. The cytotoxic effects against L929 cells of the tested SCPMs, MPMs and their respective micelleplexes determined after Trypan Blue exclusion assay, calculated as arbitrary units (AU) normalized to the number of viable cells in the untreated control (C). Statistical analysis was performed using two-way ANOVA with Dunnett's multiple comparisons test. Probability values were considered significant at * $p < 0.05$, ** $p < 0.01$ and ***$p < 0.005$ vs. control.

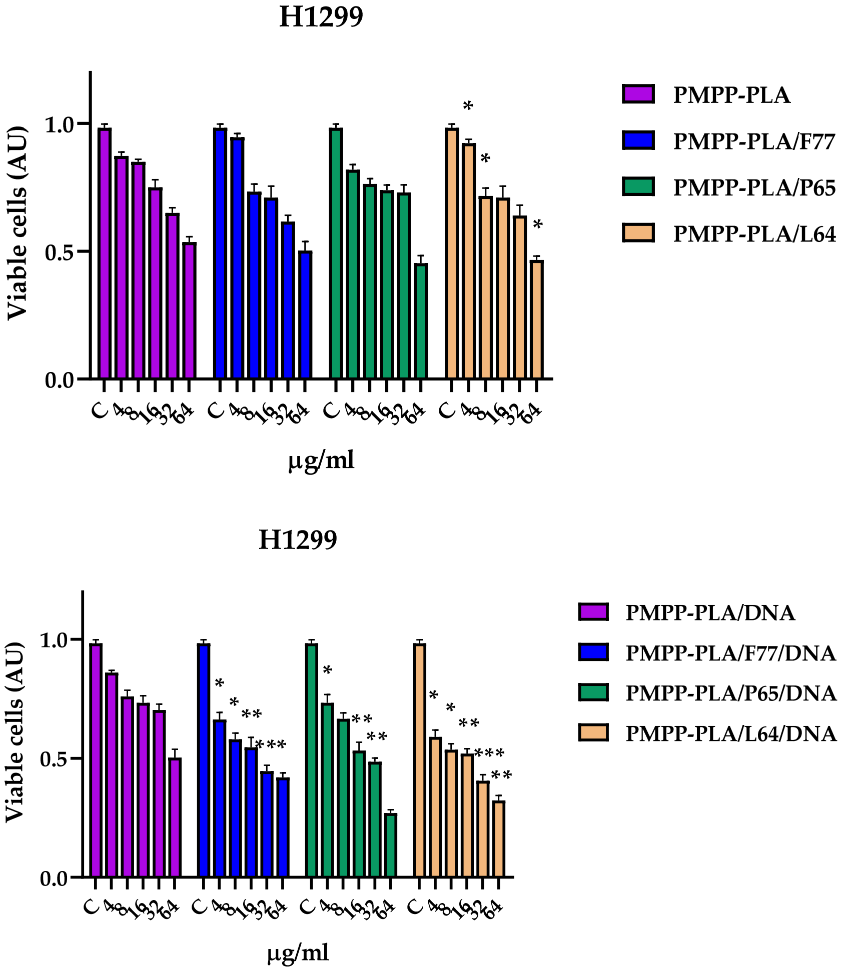

Figure S9. The cytotoxic effects against H1299 cells of the tested SCPMs, MPMs and their respective micelleplexes. The quantification of viable cells was determined after Trypan Blue exclusion assay, calculated as arbitrary units (AU) normalized to the number of viable cells in the untreated control (C). Statistical analysis was performed using two-way ANOVA with Dunnett's multiple comparisons test. Probability values were considered significant at * $p < 0.05$, ** $p < 0.01$ and ***$p < 0.005$ vs. control.

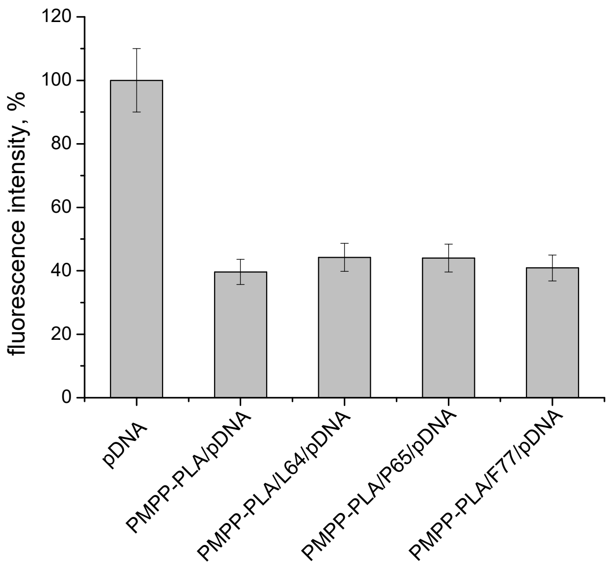

Figure S10. Ethidium bromide fluorescence quenching in micelleplexes formed from SCPMs and MPMs based on PMPP-PLA and PEO-PPO-PEO block copolymers and pDNA followed at pH 7 The fluorescence intensity of ethidium bromide in pure pDNA was taken as 100 %.

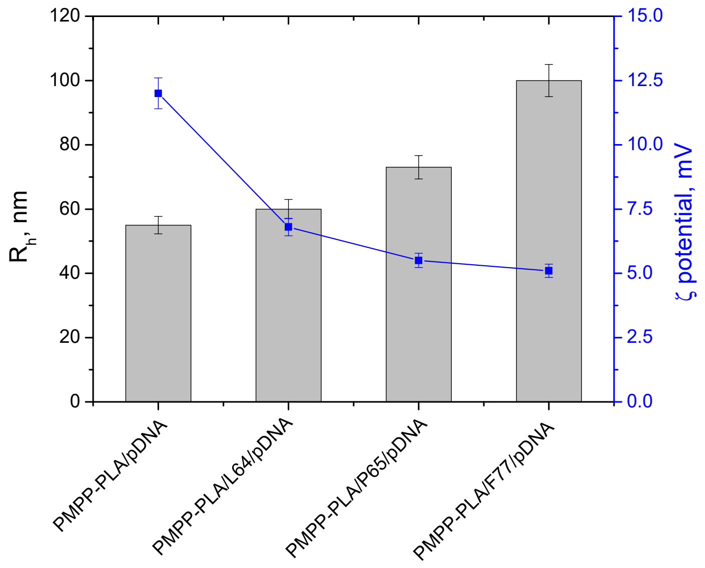

Figure S11. Hydrodynamic radius, R_h, and ζ-potential of micelleplexes formed from SCPMs and MPMs and pDNA, at N/P ratio of 10.

References:

1. Griffin WC: "Calculation of HLB Values of Non-Ionic Surfactants," Journal of the Society of Cosmetic Chemists 5 (1954): 259.
